# Transitional and CD21^−^ PD-1^+^ B cells are associated with remission in early rheumatoid arthritis

**DOI:** 10.1186/s41927-025-00487-x

**Published:** 2025-04-21

**Authors:** Sarah McGrath, Boel Sundbeck, Katrin Thorarinsdottir, Charlotte A. Jonsson, Alessandro Camponeschi, Monica Leu Agelii, Anna-Karin H. Ekwall, Merete Lund Hetland, Mikkel Østergaard, Till Uhlig, Michael Nurmohamed, Jon Lampa, Dan Nordström, Kim Hørslev-Petersen, Bjorn Gudbjornsson, Gerdur Gröndal, Ronald van Vollenhoven, Anna Rudin, Inga-Lill Mårtensson, Inger Gjertsson

**Affiliations:** 1 Department of Rheumatology and Inflammation Research, University of Gothenburg, Gothenburg, Sweden; 2 Department of Rheumatology, Sahlgrenska University Hospital, Gothenburg, Sweden; 3 Center for Rheumatology Research, Department of Rheumatology, Landspitali University Hospital, Reykjavik, Iceland; 4 Department of Clinical Immunology and Transfusion Medicine, Region Västra Götaland, Sahlgrenska University Hospital, Gothenburg, Sweden; 5 Copenhagen Center for Arthritis Research (COPECARE), Center for Rheumatology and Spine Diseases, Centre for Head and Orthopaedics, Rigshospitalet, Glostrup, Copenhagen, Denmark; 6 Department of Clinical Medicine, Faculty of Health and Medical Sciences, University of Copenhagen, Copenhagen, Denmark; 7 Division of Rheumatology and Research, Diakonhjemmet Hospital, Oslo, Norway; 8 University of Oslo, Oslo, Norway; 9 Department of Rheumatology and Clinical Immunology, Amsterdam University Medical Centers, Amsterdam, The Netherlands; 10 Amsterdam Rheumatology and Immunology Center, Reade, Amsterdam, The Netherlands; 11 Division of Rheumatology, Department of Medicine, Center for Molecular Medicine (CMM), Karolinska Institute, Stockholm, Sweden; 12 Karolinska University Hospital, Stockholm, Sweden; 13 Division of Medicine and Rheumatology, Helsinki University Hospital, Helsinki, Finland; 14 Danish Hospital for Rheumatic Diseases, University Hospital of Southern Denmark, Sønderborg, Denmark; 15 Department of Regional Health Research, University of Southern Denmark, Odense, Denmark; 16 Faculty of Medicine, University of Iceland, Reykjavik, Iceland

**Keywords:** Early rheumatoid arthritis, B cell, Transitional B cell, PD-1, Rheumatoid arthritis, Remission

## Abstract

**Background:**

Early initiation of effective treatment is associated with positive long-term prognosis for patients with rheumatoid arthritis (RA). Currently, there are no biomarkers in clinical use to predict treatment response. A predictor of treatment response may be the B-cell compartment, as this is altered in RA patients, making it a potential candidate for predicting treatment response. In this study, we sought to identify B-cell subset(s) at diagnosis that might be associated with Clinical Disease Activity Index (CDAI) remission at 24-week follow-up.

**Methods:**

Seventy early RA patients from the NORD-STAR trial, recruited from two Swedish sites, and 28 matched healthy controls, were included in this spin-off study. In NORD-STAR, all patients were randomized to methotrexate (MTX) combined with 1) prednisolone, 2) anti-TNF (certolizumab-pegol), 3) CTLA4-Ig (abatacept), or 4) anti-IL-6R (tocilizumab). Circulating B-cell subsets at diagnosis were assessed by flow cytometry. The primary outcome measure was remission according to CDAI ≤ 2.8. A multivariate two-part discriminant analysis was performed to assess whether B-cell subpopulations at diagnosis could predict remission at 24 weeks. Subsequent univariable statistical analyses were performed using t-tests, Mann-Whitney U, or Kruskal-Wallis tests, as appropriate. Correlations were analyzed using Spearman or Pearson tests, depending on data type. The impact of specific B-cell populations on remission at week 24 was assessed using logistic regression models. The logistic regression model was also used to simultaneously visualize the sensitivity and specificity of the model for all possible values of the exposure (B-cell subpopulations) in predicting the outcome.

**Results:**

Patients who achieved CDAI remission at 24 weeks had higher proportions of transitional (*p* < 0.01) and CD21^−^ PD-1^+^ (*p* < 0.01) B cells at diagnosis compared to those who did not. When the two B-cell populations were combined, the sensitivity and specificity for remission, including all treatment arms, were 59% and 86%, respectively. Stratification of the patients by treatment arm revealed a significant negative correlation between the proportion of transitional B cells at baseline and disease activity after 24 weeks of treatment with either MTX and prednisolone or anti-IL-6R.

**Conclusions:**

Our results indicate that transitional and CD21^−^ PD-1^+^ B cells are associated with remission in early RA.

**Clinical trial number:**

Not applicable.

**Supplementary Information:**

The online version contains supplementary material available at 10.1186/s41927-025-00487-x.

## Introduction

Rheumatoid arthritis (RA) is a chronic autoimmune inflammatory disease that predominantly affects the peripheral joints. The global prevalence is around 1% [1], and without effective treatment, it can result in permanent joint destruction and subsequent disability as well as pain and fatigue. The pathogenesis of RA is complex and involves the entire immune system. The role of B cells has been highlighted with autoantibodies typical for RA (rheumatoid factor (RF) and/or antibodies to citrullinated proteins (ACPAs)) appearing years before clinical onset [2, 3], and successful treatment with anti-CD20 (rituximab) B cell depletion [4]. At early disease stages, there are measurable perturbances in the peripheral blood B-cell compartment, including reduced frequencies of memory B cells, particularly unswitched memory B cells, and concomitant increased naive B cells compared to healthy individuals [5–7]. In addition, a reduction in subsets of B cells with regulatory functions (Bregs) such as transitional B cells (CD24^++^ CD38^++^), CD1d^+^ TIM-1^+^ Bregs, and IL-10 producing B10 cells has been observed in early, drug-naive RA patients [8–10].

Despite treatment advances, only 30–40% of patients respond to the first given treatment using current strategies [11]. While present prognostic factors, such as sex, high clinical disease activity, autoantibodies (RF and/or ACPAs), and early radiographic erosions, are useful in predicting treatment response at the group level, they are limited at the individual level [12, 13]. This highlights the necessity of reliable, easily determined biomarkers to predict treatment response at an individual level. Research is ongoing to explore immunophenotyping as a predictive biomarker for treatment response and stratification, which is essential also for comprehending disease mechanisms [14]. Given the functional impact of B cells and the measurable changes of this cellular compartment in the peripheral blood in early (e)RA, i.e. untreated RA patients at diagnosis, the frequency of specific B-cell populations in blood may be a suitable biomarker for treatment response. This hypothesis is supported by previous studies showing that higher frequencies of naive (IgD^+^ CD27^−^) and transitional B cells in eRA were predictive of a good response to methotrexate (MTX) [15, 16], and that good clinical response to anti-TNF and CTLA4-Ig is preceded by higher baseline levels of memory B cells [17, 18]. However, none of these findings are used in clinical routine and to the best of our knowledge, no studies have investigated the predictive potential of B-cell subset distribution in untreated eRA patients, directly comparing a range of treatment strategies including conventional synthetic (cs) and biological (b) Disease Modifying Anti-Rheumatic Drugs (DMARDs).

Our previous work has investigated an eRA cohort of 76 patients, exploring the association between B-cell subpopulations and clinical parameters at diagnosis. While CD21^−^ Double Negative (DN, IgD^−^ CD27^−^) memory B cells were associated with early radiological damage, no B cells were associated with disease activity, determined with Clinical Disease Activity Index (CDAI), at diagnosis [19]. In the present study, we therefore investigated if any B-cell subsets in the NORD-STAR treatment trial of drug-naive eRA patients could predict treatment response to different first-line strategies at 24-week follow-up. To this end, we examined B-cell subsets at diagnosis and their association to treatment response to MTX combined with 1) prednisolone, 2) anti-TNF, 3) CTLA4-Ig, or 4) anti-IL-6R.

## Materials and methods

### Patients and healthy controls

This study was conducted on a subset of *n* = 76 patients with newly diagnosed RA, according to the American College of Rheumatology/European League Against Rheumatism 2010 criteria from the NORD-STAR cohort [19–21]. Before starting treatment, blood samples were taken. B-cell data at diagnosis and disease activity assessment at the 24-week follow-up were available for *n* = 70 patients, hence further analysed in the present study. Patients were compared with a group of *n* = 28 age- and sex-matched healthy controls (HC).

The complete protocol and results of the primary outcomes at week 24 of the NORD-STAR trial are published [20], in brief patients in Sweden were randomised to one of four treatment arms: MTX and prednisolone, MTX and certolizumab-pegol (anti-TNF; UCB, Brussels, Belgium), MTX and abatacept (CTLA4-Ig; Bristol Myers Squibb, New York City, NY, USA), MTX and tocilizumab (anti-IL-6R; Hoffman-La Roche, Basel, Switzerland). MTX was escalated to 25 mg/week within the first four weeks. Patients receiving prednisolone were tapered from 20 mg/day to 5 mg/day within nine weeks and discontinued after 36 weeks.

All patients signed the informed consent form, and the study was approved by the regional ethics committees of Gothenburg and Lund, Sweden (691-12, 2012-10-03 and T270-13, 2013-04-08). For the HC, no personal information or identity was recorded and no written consent or approval by the Human Research Ethics Committee was required (Swedish law 2003: 460, paragraphs 4 and 13). The study was conducted in compliance with the Declaration of Helsinki.

### Clinical disease assessments

As described previously [20] disease activity was assessed using the Disease Activity Score of 28 joints (DAS28) at diagnosis and the Clinical Disease Activity Index (CDAI) and their components at diagnosis and at 24 weeks after treatment initiation. The primary clinical outcome was remission, defined as CDAI ≤ 2.8. DAS28, which includes C-Reactive Protein (CRP) or erythrocyte sedimentation rate (ESR), could be influenced by the IL-6 inhibitor and was only assessed at diagnosis.

Seropositivity was determined at the time of diagnosis. ACPA, determined as anti-CCP, and RF were determined at the Laboratory for Clinical Immunology at Sahlgrenska University [19]. Patients with ≥20 IU/ml serum ACPA or RF were considered ACPA or RF positive [19, 20, 22].

### Flow cytometry

Peripheral blood was acquired at diagnosis, plasma was separated from the cellular compartment via centrifugation (800 g, 10 minutes) and stored at –80 °C. Peripheral blood mononuclear cells (PBMCs) were isolated, blocked, stained and acquired according to previously published protocol [19, 23]. Antibodies and dilutions used in flow cytometry analysis are shown in Suppl. Table 1. Data were analysed using Flow Jo software (Tree Star Ashland, OR, USA).

### Gating strategy for flow cytometry

B cells were identified in PBMCs as single lymphocytes expressing CD19. Co-expression of CD24 and CD38 within CD19^+^ B cells was used to determine transitional B cells (CD24^++^ CD38^++^), naive B cells (CD24^+^ CD38^+^), and memory B cells (CD24^+/lo^ CD38^lo^). CD19^+^ B cells were also divided according to their expression of the CD21 co-receptor into CD21^+^ and CD21^−^ populations. Gating on CD27 vs IgD, we defined a further four B-cell subsets: naive and transitional cells (NAV, CD27^−^ IgD^+^), switched memory B cells (SW MBCs, CD27^+^ IgD^−^), unswitched MBCs (UnSW, CD27^+^ IgD^+^), and double negative MBCs (DN, CD27^−^ IgD^−^); these populations were identifiable in both CD21^+^ and CD21^−^ populations. Within the CD21^−^ population, plasmablasts were identified as CD38^++^ CD24^−/lo^ and CD21^−^B cells were also divided according to their expression of PD-1. Representative eRA sample of the gating strategy shown in Suppl. Fig. 1.

### Statistical analysis

Two-class discriminant analysis (OPLS-DA) was used to examine whether B-cell subpopulation frequencies at diagnosis could discriminate patients who reached disease remission after 24 weeks of treatment from those who did not. Data were first normalized using a log transformation and were further scaled to unit variance (by dividing each variable by its standard deviation) so that all the variables were given an equal weight regardless of their absolute value. The loading vectors were normalized to length 1. The OPLS model performance was assessed according to R2 (amount of variation explained) and Q2 (how well the outcome can be predicted by the model in a cross-validation sample). In an OPLS-DA loading plot, the X–variables extend in either a positive or negative direction to illustrate their association with the binary Y-variable. These statistical analyses were conducted in SIMCA version 17.0.1; Umetrics, Umea, Sweden. Subsequent univariable statistical analyses were performed using unpaired T-test for normally distributed data, otherwise Mann-Whitney U-test followed by Bonferroni-Dunn correction for multiple testing where appropriate. Kruskal-Wallis test for ≥2 groups for continuous variables, followed by Dunn’s multiple comparison test where appropriate. Correlation analyses were performed by Spearman’s Rank correlation or Pearson correlation test. The impact of specific B-cell populations on remission at week 24 (outcome of interest) was assessed in logistic regression models. From the logistic model we obtained receiver operating characteristic curves (ROC), which are a standard way to visualize simultaneously the sensitivity and specificity of the model for all possible values of the exposure (B-cell subpopulations) for predicting the outcome. Cut-off values were selected on the basis of achieving a high specificity (80–90%) while maintaining a reasonable trade-off in sensitivity. The positive and negative predictive values (PPV and NPV respectively) were calculated based on the remission rate. Differences in demographic factors between HC and eRA, as well as differences in both demographic factors and clinical outcomes among eRA patients stratified by treatment arm, were assessed according to Mann-Whitney U test (Kruskal-Wallis test for ≥2 groups for continuous variables) or Chi Squared test (Fisher’s exact test for small samples, dichotomous endpoints). Statistical analyses were performed using GraphPad Prism software (GraphPad Prism software la Jolla, CA, USA) and SPSS software (IBM SPSS Statistics, Armonk, NY, USA).

## Results

### Characteristics of the study subjects

Demographics and clinical characteristics of the cohort of eRA patients at diagnosis are shown in Table 1. There were no significant differences in age or sex between HC and eRA patients or between patients in the different treatment arms (Table 1). The number of tender and swollen joints, the composite measures scores of disease activity (DAS28 and CDAI), and the number of patients positive for ACPA and/or RF did not differ significantly between treatment arms. As previously reported, in this spin-off study, CRP levels at diagnosis, were significantly lower in patients randomized to anti-IL-6R treatment compared to patients in the anti-TNF treatment [21]. At the 24-week follow-up, the median CDAI was 3.8, with 40% of patients achieving CDAI remission. In this subgroup no significant difference was observed in the proportions of patients who achieved remission across the various treatment arms (Table 2). Disease activity at diagnosis was similar across treatment arms when stratified by remission status at the 24-week follow-up (Suppl. Table 2).

**Table 1:** Cohort demographics and baseline clinical observations

	HC (*n* = 28)	eRA (*n* = 70)	*p*-value	MTX + prednisolone (*n* = 12)	MTX + anti-TNF (*n* = 22)	MTX + CTLA4-Ig (*n* = 17)	MTX + Anti-IL-6R (*n* = 19)	*p*-value
Age, yr^a^	59.5 (20–75)	57 (21–80)	0.98^h^	60 (24–80)	61 (21–71)	61 (21–77)	51 (25–72)	0.43^f^
Female, *n* (%)	17 (61)	49 (70)	0.48^i^	10 (83)	13 (59)	12 (71)	14 (74)	0.53^g^
Smoker, *n* (%)^e^	ND	11 (16)		1 (8.3)	4 (18)	2 (12)	4 (21)	0.84^g^
Symptom duration, months^a, b^	NA	5 (1–23)		4 (1–11)	5 (1–18)	6 (2–23)	6 (1.5–21)	0.57^f^
CRP, mg/L^a^	ND	9 (0.3–180)		15 (2–152)	19 (2–180)	10 (2–92)	5.4 (0.3–22)	0.035^*f^
ESR, mm/hr^a^	ND	26.5 (5–108)		29 (5–108)	32 (7–98)	28 (8–101)	19 (5–37)	0.08^f^
SJC66^a^	NA	11 (3–30)		18 (4–30)	12 (3–28)	8 (3–19)	12 (3–17)	0.09^f^
TJC68^a^	NA	14 (2–47)		18 (6–34)	15 (2–35)	13 (3–35)	12 (3–47)	0.28^f^
SJC28^a^	NA	8 (2–24)		13 (4–22)	8 (3–24)	6 (3–14)	9 (2–13)	0.13^f^
TJC28^a^	NA	9 (0–27)		10 (3–21)	8 (1–27)	9 (0–13)	8 (0–24)	0.44^f^
DAS28-CRP^a^	NA	5.0 (2.7–8.3)		5 (3.6–7.7)	5.4 (3.2–8.3)	4.9 (3.8–6.5)	4.7 (2.7–6.9)	0.18^f^
DAS28-ESR^a^	NA	5.3 (2.6–8.7)		5.6 (3.7–8.2)	5.8 (3.6–8.7)	5.2 (4.2–7.2)	5.2 (2.6–7.1)	0.15^f^
CDAI^a^	NA	27.9 (10.1–68.7)		32 (13.3–56.9)	28.1 (10.1–68.7)	26 (14.3–41.7)	27.1 (10.5–52.5)	0.4^f^
ACPA^+^, n (%)^c^	NA	58 (83)		10 (83)	17 (77)	15 (88)	16 (84)	0.9^g^
RF^+^, n (%)^d^	NA	52 (74)		10 (83)	13 (59)	12 (71)	17 (90)	0.14^g^
ACPA^+^ RF^+^, n (%)^c, d^	NA	47 (67)		9 (75)	13 (59)	11 (65)	14 (74)	0.71^g^
ACPA^−^ RF^−^, n (%)^c, d^	NA	7 (10)		1 (8.3)	5 (23)	1 (6)	0 (0)	0.09^g^

^a^Median and range

^b^Retrospective patient-reported pain in joints before RA diagnosis

^c^Patients with ACPA levels ≥20IU/ml are considered ACPA+

^d^Patients with RF levels ≥20 IU/ml are considered RF+

^e^Current daily smoker

^f^Difference between treatment arms, Kruskal-Wallis test *p < 0.05

^g^Difference between treatment arms, Fisher’s exact test

^h^Difference between HC and eRA, Mann-Whitney U-test

^i^Difference between HC and eRA, Chi-Square test

ND: Not Detected; NA: Not Applicable; HC: Healthy Control; eRA: early Rheumatoid Arthritis; CRP: C Reactive Protein; ESR: Erythrocyte Sedimentation Rate; SJC: Swollen Joint Count; TJC: Tender Joint Count; DAS28: Disease Activity Score for 28 joints; CDAI: Clinical Disease Activity Index; ACPA: Anti-Citrullinated Protein antibodies; RF: Rheumatoid Factor

**Table 2:** Clinical outcomes in eRA cohort after 24 weeks of treatment

	eRA all treatment arms (*n* = 70)	MTX + prednisolone (*n* = 12)	MTX + anti-TNF (*n* = 22)	MTX + CTLA4-Ig (*n* = 17)	MTX + anti-IL-6R (*n* = 19)	*p*-value
CDAI Remission, n (%)	28 (40)	2 (16.7)	8 (36.4)	10 (59)	8 (42)	0.16^b^
CDAI^a^	3.8 (0.1–28.3)	5 (1.6–15.5)	4.2 (0.2–19.2)	2.3 (0.1–28.3)	3.3 (0.1–18.5)	0.26^c^
TJC 28^a^	0.5 (0–19)	1 (0–11)	0 (0–4)	0 (0–19)	1 (0–15)	0.26^c^
SJC 28^a^	0.0 (0–6)	0 (0–1)	0 (0–6)	0 (0–6)	0 (0–3)	0.61^c^
PGA^a^	14.5 (0–92)	19 (2–62)	14.5 (2–92)	9 (0–60)	10 (0–76)	0.099^c^
EGA^a^	6.5 (0–40)	11 (1–34)	7.5 (0–40)	5 (0–24)	4 (0–22)	0.33^c^

^a^Median and range

^b^Difference between treatment arms, Fisher’s exact test

^c^Difference between treatment arms, Kruskal-Wallis test

eRA: early Rheumatoid Arthritis; CDAI: Clinical Disease Activity Index; SJC: Swollen Joint Count; TJC: Tender Joint Count; PGA: Patient Global Assessment; EGA: Evaluator Global Assessment

### A higher proportion of transitional and CD21^−^ PD-1^+^ B cells at diagnosis is associated with follow-up CDAI remission

**Fig. 1:**
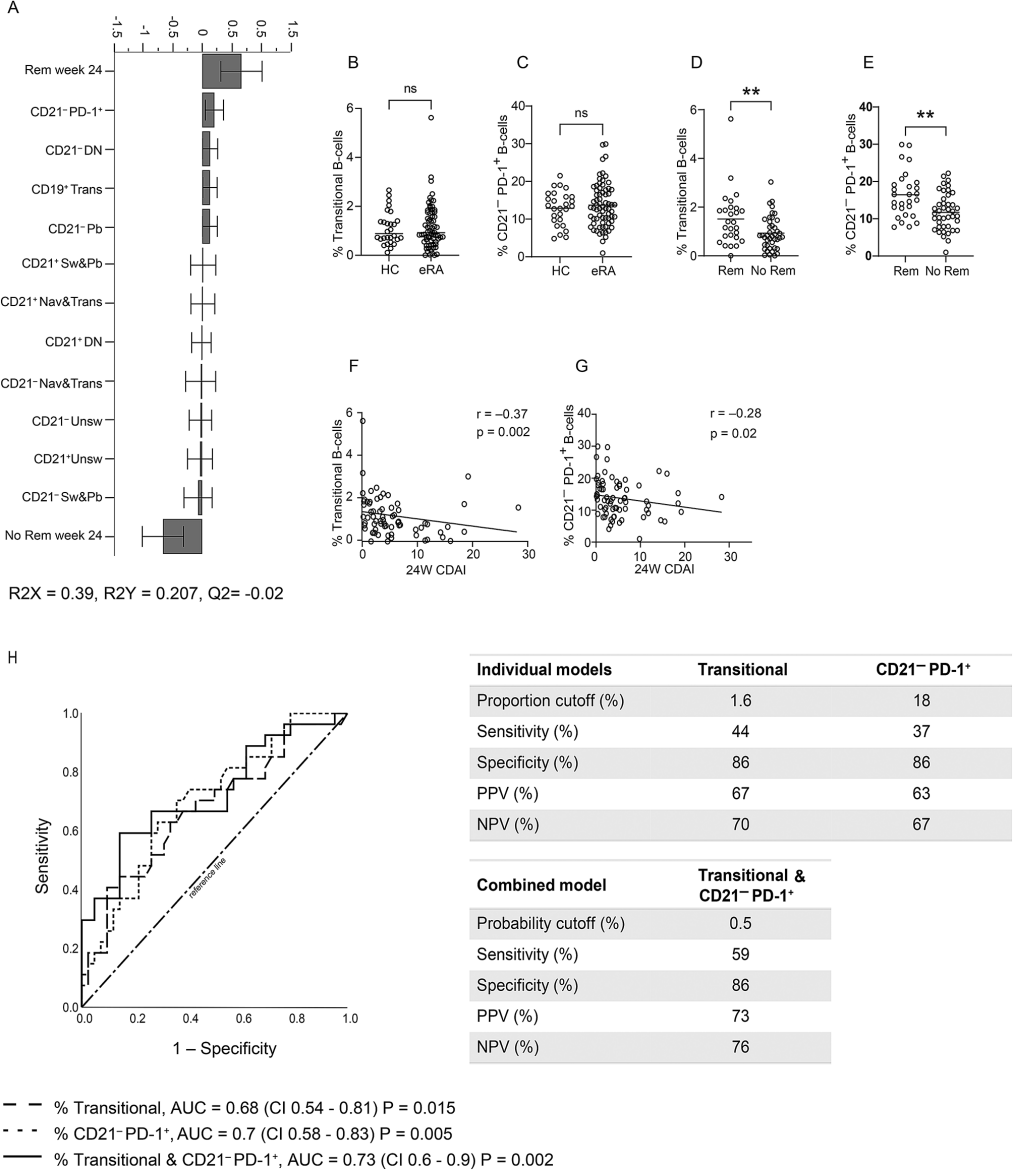
Transitional and PD-1^+^ B cells in eRA patients at diagnosis and association with 24-week remission. (**A**) OPLS-DA loading plot of the association between B-cell subset frequencies at diagnosis (X–variables) and remission status after 24 weeks of treatment (binary Y-variables). (**B**) Frequency of transitional B cells (CD24^++^CD38^++^; in total B cells) in HC (N = 28) and eRA patients (N = 69) at diagnosis, Mann-Whitney U-test. (**C**) Frequency of PD-1^+^ B cells (of parent population CD21^−^ B cells) in HC (N = 27) and in eRA patients (N = 70) at diagnosis, unpaired T-test. (**D**) Frequency of transitional B cells in eRA patients at diagnosis, subdivided by 24-week follow-up remission status, unpaired T-test. (**E**) Frequency of CD21^−^ PD-1^+^ B cell, in eRA patients at diagnosis, subdivided by 24-week follow-up remission status, unpaired T-test. (**F**) Scatter plot and Spearman’s Rank correlation analyses of transitional B-cell frequency at diagnosis against 24-week follow-up CDAI. (**G**) Scatter plot and Spearman’s Rank correlation analyses of CD21^−^ PD-1^+^ B-cell frequency at diagnosis against 24-week follow-up CDAI. (**E**) ROC curves plotting individual regression models of transitional and CD21^−^ PD-1^+^ B-cell frequencies at diagnosis (dashed and dotted lines, respectively) or combined in one multiple regression model (black line). In panel B, the horizontal line indicates the median value, while in panels C– E horizontal lines indicate the mean values. ** *p* < 0.01. DN: Double Negative; Trans: Transitional; Pb: Plasmablast; Sw: Switched; Nav: Naive; Unsw: Unswitched; ns: non-significant; HC: Healthy Control; eRA: early Rheumatoid Arthritis; Rem: Remission; CDAI: Clinical Disease Activity Index; ROC: Receiver Operating Characteristic; AUC: Area Under the Curve; CI: 95 percent confidence interval; PPV: Positive Predictive Value; NPV: Negative Predictive Value

First, we investigated whether any circulating B-cell subpopulations at diagnosis could predict CDAI remission at 24-week follow-up. Multivariate discriminant analysis (OPLS-DA) revealed four B-cell subpopulations that were positively associated with remission: CD21^−^ PD-1^+^, CD21^−^ DN, transitional B cells and CD21^−^ plasmablasts (Pb), while no subpopulations were related to lack of remission (Fig. 1A, gating strategy in Suppl. Fig. 1).

Subsequently, the potential influence of demographic or clinical factors on these B-cell subpopulations was assessed (Suppl. Table 3). As expected, there was a significant positive correlation between CD21^−^ DN B cells and age [19], and RF positivity was associated with a lower proportion of CD21^−^ DN. The frequency of transitional B cells at diagnosis correlated negatively with disease duration. Potential confounders, such as age, symptom duration, and RF, were considered. However, these factors were not true confounders, as they were associated only with the predictors of interest (exposure) and not with the outcomes of interest (Suppl. Table 4). Therefore, adjusting the analyses for these factors could introduce overadjustment bias, leading to a loss of accuracy and precision in estimating the exposure-outcome relationship. None of the four B-cell populations correlated with CRP at diagnosis or at week 24 (Suppl. Table 3). CRP at diagnosis was not associated with remission at the 24-week follow-up, even when the anti-IL-6R group, which exhibited lower CRP, was excluded (Suppl. Table 4). Overall, no confounding demographic or clinical variables were identified.

At diagnosis, there were no significant differences between HC and eRA in the proportions of transitional or CD21^−^ PD-1^+^ B cells in univariable analyses (Fig. 1B and C). However, within the eRA cohort, the frequencies of these populations were significantly higher in patients who reached remission (Fig. 1D and E). Moreover, the frequency of both B-cell subpopulations correlated negatively with CDAI at follow-up (Fig. 1F and G). At diagnosis, the frequency of CD21^−^ plasmablasts was significantly higher in eRA than in HC and in patients who reached remission at 24-week follow-up, but did not correlate with follow-up CDAI (Suppl. Fig. 2A-C). There were no significant differences in the proportions of CD21^−^ DN at diagnosis between HC and eRA (Suppl. Fig. 2D). Furthermore, the proportions of CD21^−^ DN at diagnosis were not associated with remission and there was no correlation with follow-up CDAI (Suppl. Fig. 2E and F). As only transitional and CD21^−^ PD-1^+^ B cells correlated with follow-up CDAI, we proceeded to test the predictive ability of these two subsets for follow-up response using logistic regression presented as a ROC curve. Both populations predicted remission with an AUC of 0.68 (95% CI 0.54–0.81) and 0.7 (95% CI 0.58–0.83), respectively (Fig. 1H). Combining these two populations into a single model, AUC was 0.73 (CI 0.6–0.9), whereas sensitivity increased from 44% and 37% to 59% with preserved specificity of 86% and both the PPV and NPV increased to 73% and 76% respectively (Fig. 1H). In summary, the frequencies of circulating transitional and CD21^−^ PD-1^+^ B cells at diagnosis were significantly elevated in patients who reached CDAI remission at 24-week follow-up.

### A higher frequency of transitional and CD21^−^ PD-1^+^ B cells correlate with a lower global health score at follow-up and the absence of tender joints

**Fig. 2:**
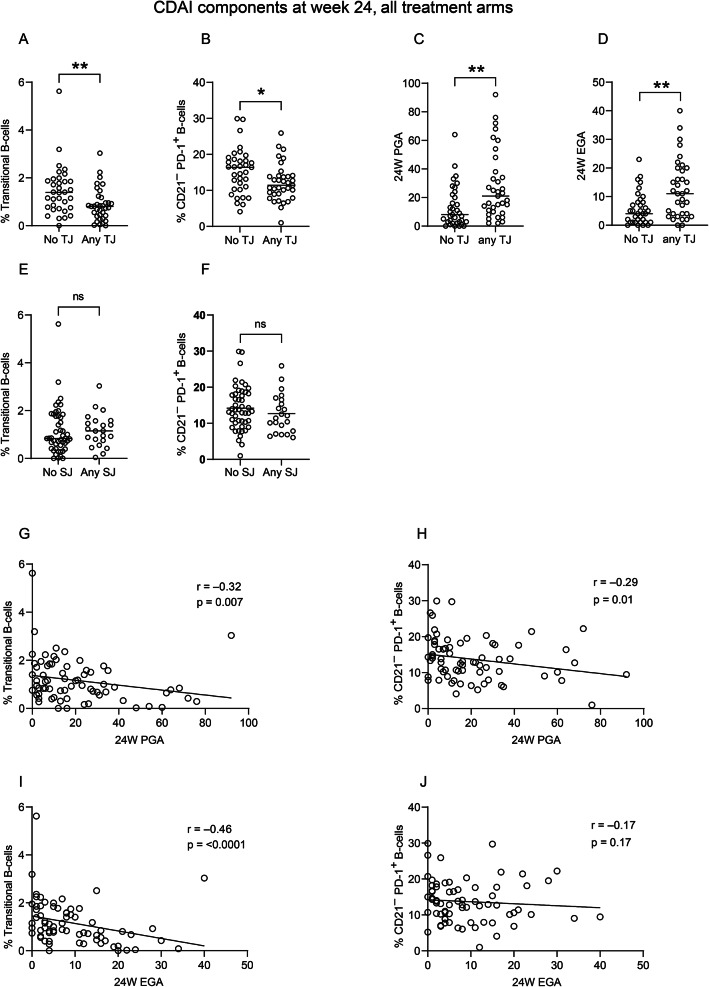
Transitional and CD21^−^PD-1^+^B cells in eRA patients at diagnosis and association with 24-week CDAI components. (**A**) Frequency of transitional B cells (CD24^++^CD38^++^; in total B cells) in eRA patients (N = 69) at diagnosis, subdivided by 24-week follow-up tender joints, Mann-Whitney U-test. (**B**) Frequency of PD-1^+^ B cells (of parent population CD21^−^ B cells) in eRA patients (N = 70) at diagnosis, subdivided by 24-week follow-up tender joints, Mann-Whitney U-test. (**C**) PGA in eRA patients (N = 70) at 24-week follow up, subdivided by 24-week follow-up tender joints, Mann-Whitney U-test. (**D**) EGA in eRA patients at 24-week follow up, subdivided by 24-week follow-up swollen joints, Mann-Whitney U-test. (**E**) Frequency of transitional B cells in eRA patients at diagnosis, subdivided by 24-week follow-up swollen joints, Mann-Whitney U-test. (**F**) Frequency of CD21^−^ PD-1^+^ B cells, in eRA patients at diagnosis, subdivided by 24-week follow-up swollen joints, unpaired T-test. (**G**) Scatter plot and Spearman’s Rank correlation analyses of transitional B-cell frequency at diagnosis against 24-week follow-up PGA. (**H**) Scatter plot and Spearman’s Rank correlation analyses of CD21^−^ PD-1^+^ B-cell frequency at diagnosis against 24-week follow-up PGA. (**I**) Scatter plot and Spearman’s Rank correlation analyses of transitional B-cell frequency at diagnosis against 24-week follow-up EGA. (**J**) Scatter plot and Spearman’s Rank correlation analyses of CD21^−^ PD-1^+^ B-cell frequency at diagnosis against 24-week follow-up EGA. In panels A–E, horizontal lines indicate the median values, while in panel F, the horizontal line indicates the mean value *p < 0.05, ** *p* < 0.01; ns: non-significant; eRA: early Rheumatoid Arthritis; TJ: Tender Joints; SJ: Swollen Joints; PGA: Patient Global Assessment; EGA: Evaluator Global Assessment

Next, we sought to determine which CDAI components at follow-up contributed to the association between remission and the frequencies of transitional and CD21^−^ PD-1^+^ B cells at diagnosis. Patients with no tender joints at follow-up had a higher proportion of transitional and CD21^−^ PD-1^+^ B cells at diagnosis compared to patients with any tender joint(s) (Fig. 2A and B). As anticipated, these patients also scored lower for both PGA and EGA (Fig. 2C and D). There were no significant associations between these B-cell populations and the number of swollen joints (Fig. 2E and F), although they correlated negatively with PGA at week 24 (Fig. 2G and H). The frequency of transitional B cells, but not CD21^−^ PD-1^+^ B cells correlated significantly with EGA (Fig. 2I and J).

In summary, our findings suggest that a higher proportion of transitional and CD21^−^ PD-1^+^ subpopulations at diagnosis is associated with the absence of tender joints and reduced patient- and evaluator global health scores at follow-up.

### Higher proportion of transitional B cells at diagnosis is associated with lower disease activity in response to methotrexate combined with prednisolone or anti-IL-6R therapy

**Fig. 3:**
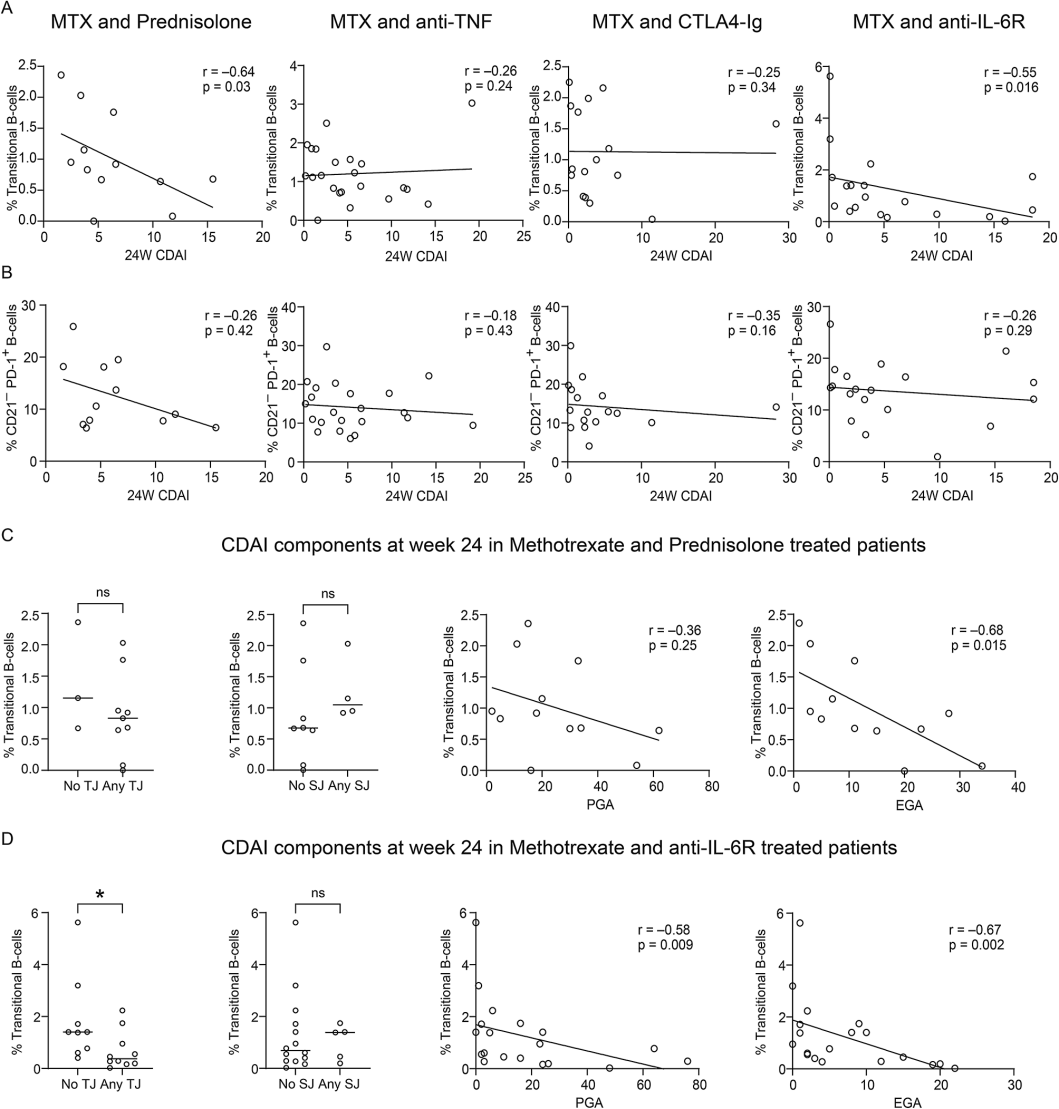
Transitional and CD21^−^PD-1^+^B cells in eRA at diagnosis and association with 24-week CDAI, by treatment. (**A**) Scatter plot and Spearman’s Rank correlation analyses of transitional B-cell (CD24^++^CD38^++^; in total B cells) frequency at diagnosis against 24-week follow-up CDAI in the following treatment arm; MTX and Prednisolone (N = 12), MTX and anti-TNF (N = 22), MTX and CTLA4-Ig (N = 16), MTX and anti-IL-6R (N = 19). (**B**) Scatter plot and Spearman’s Rank correlation analyses of CD21^−^ PD-1^+^ B-cell (of parent population CD21^−^ B cells) frequency at diagnosis against 24-week follow-up CDAI in the following treatment arm; MTX and Prednisolone (N = 12), MTX and anti-TNF (N = 22), MTX and CTLA4-Ig (N = 17), MTX and anti-IL-6R (N = 19). (**C**) Frequency of transitional B cells in eRA patients at diagnosis, subdivided by 24-week follow-up CDAI components in MTX and Prednisolone treatment arm, Mann-Whitney U-test and Pearson test. (**D**) Frequency of transitional B cells in eRA patients at diagnosis, subdivided by 24-week follow-up CDAI components in MTX and anti-IL-6R treatment arm, Mann-Whitney U-test and Spearman’s Rank correlation analyses. Horizontal lines indicate the median value. *p < 0.05; ns: non-significant. eRA: early Rheumatoid Arthritis; MTX: Methotrexate; anti-TNF: (certolizumab-pegol); CTLA4-Ig (abatacept); anti-IL-6R: (tocilizumab); CDAI: Clinical Disease Activity Index

Patients were randomised to one of four treatments arms combining MTX with: 1) prednisolone, 2) certolizumab-pegol (anti-TNF), 3) abatacept (CTLA4-Ig), or 4) tocilizumab (anti-IL-6R). These four treatments target different immunological pathways and the effects on B-cell populations may therefore vary depending on treatment. Considering this, we investigated correlations between transitional and CD21^−^ PD-1^+^ B cells at diagnosis and CDAI at 24-week follow-up within each treatment arm. A negative correlation between the proportion of transitional B cells, but not CD21^−^ PD-1^+^ B cells, at diagnosis and CDAI at week 24 was found only in patients treated with MTX combined with prednisolone or anti-IL-6R (Fig. 3A and B). The proportion of transitional B cells did not differ between the different treatment arms at diagnosis (Suppl. Figure 3 and Suppl. Table 5). Furthermore, in patients treated with MTX and prednisolone, the frequency of transitional B cells was not associated with the number of tender or swollen joints, or with PGA, but correlated significantly with EGA (Fig. 3C). However, in patients treated with MTX in combination with anti-IL-6R, the frequency of transitional B cells at diagnosis was significantly associated with tender joints, PGA score, and EGA score at 24-week follow-up (Fig. 3D).

No significant correlations were found between CDAI at 24-week follow-up and frequencies of CD21^−^ plasmablasts nor CD21^−^ DN at diagnosis when patients were divided by treatment arm (Suppl. Fig. 4A and B). There were no significant differences in the median proportions of the four B-cell subpopulations of interest between remission and non-remission patients across treatment arms at 24 weeks, likely due to the limited sample size in each group (Suppl. Fig. 4C).

Overall, a higher proportion of transitional B cells at diagnosis was associated with improved response to MTX in combination with either prednisolone or anti-IL-6R treatment. This indicates the potential for transitional B cells to be predictive of the response to a particular treatment.

## Discussion

In this study, we investigated whether circulating B-cell subpopulations in eRA were associated with 24-week treatment response in a subset of a four-armed randomised trial with active conventional treatment or three different biological agents. In all patients, without stratifying by treatment arm, the frequency of both transitional and CD21⁻ PD-1⁺ B cells at diagnosis was found to be elevated in those who achieved remission within 24 weeks. According to ROC curve prediction modelling, these B-cell populations suggest future remission, particularly in relation to patient subjective symptoms. The frequency of transitional B cell correlates specifically with response to MTX and prednisolone and MTX combined with anti-IL-6R.

A higher frequency of transitional B cells in patients achieving good treatment response to csDMARDs in our study is supported by previous reports [16, 24]. However, our study is the first to identify a higher frequency of transitional B cells at diagnosis preceding remission in response to csDMARDs in combination with glucocorticoids and biologics, in particular anti-IL-6R treatment. B cells both respond to, and are major producers of, IL-6, and B cell over-production of IL-6 is known to contribute to autoimmunity, through spontaneous germinal centre formation [25–27]. Previous work identified a clinically good response after 3 months of anti-IL-6R treatment coinciding with an expansion of CD25^high^ B cells producing TGF-β, here defined as Bregs, and a reduction in activated B cells [28]. In various autoimmune conditions including systemic lupus erythematosus and systemic sclerosis, transitional B cells produce excessive amounts of IL-6, driven by type-1 interferon and toll-like receptor-7 activation [29–31]. Thus, it is also possible that the IL-6R antagonism in RA inhibits the function of IL-6 derived from transitional B cells.

Transitional B cells are a heterogeneous population known to contain Bregs. Although the definition of Bregs varies [32] they are mainly associated with IL-10 production, and enrichment of Bregs has been repeatedly observed in the transitional (CD24^++^ CD38^++^) B-cell compartment [8, 16, 32–34]. Bregs function by suppressing T-cell activation via several mechanisms: for example IL-10 production, and by their expression of PD-L1 [35–37].

We observed an increased frequency of CD21^−^ PD-1^+^ B cells in patients who achieved remission. However, CD21^−^ PD-1^+^ B cells were not predictive of response to any specific treatment. The PD-1/PD-L1 axis is a regulatory pathway, that prevent the activation of T-cells during antigen presentation [38]. Much is known about the inhibitory function of PD-1 on T-cells and the importance of this in the pathogenesis of RA [39–41]. Somewhat in agreement with our findings, PD-1 expression levels on T-cells are lower in RA patients and inversely correlate with disease activity [21]. The function of PD-1 in B cells is not yet fully understood, but activation of PD-1 inhibits B-cell receptor signalling, and thus regulates B cell activation [42]; however there is also evidence that PD-1 B cells accumulate in the inflamed joint and contribute to disease pathogenesis in RA [43]. PD-1 inhibition in checkpoint immune therapy can lead to immune-related adverse events including development of arthritis [44]. Our results might suggest that these PD-1^+^ B cells are part of regulatory circuits that with the appropriate treatment may succeed in controlling the aberrant autoimmune responses. This becomes even more interesting since several PD-1-agonists are being developed as new treatments for RA, e.g. peresolimumab [45].

This study is hypothesis-generating with the strengths that it is performed in a blinded randomised trial with a well-characterised cohort of early, drug-naive RA patients, which allows for the direct comparison between four treatment arms and well-characterised B-cell subsets. A limitation of this study is the small number of patients in each treatment arm, which may affect the statistical power for subgroup analyses. Additionally, non-remission is a multifactorial outcome influenced by variables beyond B-cell subsets, including treatment adherence, pharmacokinetics, and comorbidities, none of which were accounted for in this study. Implementing this methodology in clinical practice also presents challenges, including sample handling, processing delays, and the need for specialized equipment. While such infrastructure is not universally available, a strength of this study is that all samples were processed from fresh blood in a dedicated clinical immunology laboratory, ensuring high sample quality. Furthermore, reliance on flow cytometry alone limits the ability to fully capture the heterogeneity and function of B-cell subsets.

## Conclusion

The findings of this exploratory study indicate that transitional and CD21^−^PD-1^+^ B cells are associated with remission in eRA. The study emphasises the necessity of analysing subsets of B cells in order to gain insight into their role in the immunoregulation and pathogenesis of RA, and to understand how they may contribute to responses to different treatment strategies.

## Electronic supplementary material

Below is the link to the electronic supplementary material.

Supplementary Material 1 Supplemental Table 1: Antibodies for Flow Cytometry Staining

Supplementary Material 2 Supplemental Table 2: Cohort demographics and baseline clinical observations subdivided by 24-week follow-up remission status and treatment arm

Supplementary Material 3 Supplemental Table 3: Demographic and clinical confounding variables with B cell populations of interest at diagnosis

Supplementary Material 4 Supplemental Table 4: Demographic and clinical confounding variables association with treatment response

Supplementary Material 5 Supplemental Table 5: Statistical analysis of transitional B Cells in eRA patients at diagnosis across treatments

Supplementary Material 6. Supplementary Figures

## Data Availability

The datasets used and/or analysed the current study are available from the corresponding author on reasonable request.

## Abbreviations

ACPA: Anti-Citrullinated Protein Antibody
AUC: Area Under the Curve
Breg: Regulatory B cell
CDAI: Clinical Disease Activity Index
CRP: C-reactive Protein
csDMARD: Conventional Synthetic Disease Modifying Anti-Rheumatic Drug
CTLA4: Cytotoxic T-lymphocyte Antigen 4
DAS28: Disease Activity Score, including a 28-joint count
DMARD: Disease Modifying Anti-Rheumatic Drug
DN: Double Negative
EGA: Evaluator Global Assessment
eRA: Early Rheumatoid Arthritis
ESR: Erythrocyte Sedimentation Rate
HC: Healthy Control
Ig: Immunoglobulin
IL-6R: IL-6 Receptor
MBC: Memory B cells
MTX: Methotrexate
NA: Not Applicable
NAV: Naive and transitional B cells
ND: Not Detected
NPV: Negative Predictive Value
NS: Non-Significant
OPLS-DA: Orthogonal Projections to Latent Structures Discriminant Analysis
Pb: Plasmablast
PBMC: Peripheral Blood Mononuclear Cells
PD-1: Programmed cell Death protein 1
PD-L1: Programmed Death Ligand 1
PGA: Patient Global Assessment
PPV: Positive Predictive value
RA: Rheumatoid Arthritis
REM: Remission
RF: Rheumatoid Factor
ROC: Receiver Operating Characteristic
SJC: Swollen Joint Count
Sw: Switched
ΤGF–β: Transforming Growth Factor–Beta
TIM-1: T-cell Ig and mucin domain protein 1
TJC: Tender Joint Count
TNF: Tumour Necrosis Factor
UnSW: Unswitched
VAS: Visual Analogue Scale
