# Supplementary material for: Transitional and CD21− PD-1+ B cells are associated with remission in early rheumatoid arthritis
Source: BMC Rheumatol. 2025 Apr 21;9:45. doi: 10.1186/s41927-025-00487-x (PMC12010607; doi:10.1186/s41927-025-00487-x)
Supplement: Supplementary file 1 — Supplementary Material 1 Supplemental Table 1: Antibodies for Flow Cytometry Staining [file 41927_2025_487_MOESM1_ESM.docx]

**Supplemental Table 1. Antibodies for Flow Cytometry Staining**

| Antibody Clone | Company | Dilution |
| --- | --- | --- |
| CD19-V500 HIB19 | BD Horizon | 40 |
| CD21-PECy5 B-ly4 | BD Pharmingen | 40 |
| CD23-APC M-L233 | BD Pharmingen | 80 |
| CD24-PECy7 ML5 | BD Pharmingen | 6.67 |
| CD38-BV421 HIT2 | BD Horizon | 6.67 |
| CD27-PE L128 | BD | 40 |
| IgD-FITC IA6-2 | BD Pharmingen | 40 |
| IgG-PECy7 G18-145 | BD Pharmingen | 25 |
| PD-1-PE EH12.2H7 | Biolegend | 100 |
| PD-L1-BV421 29E.2A3 | Biolegend | 50 |
| CD4-FITC RPA-T4 | BD Pharmingen | 25 |
