# Supplementary material for: Transitional and CD21− PD-1+ B cells are associated with remission in early rheumatoid arthritis
Source: BMC Rheumatol. 2025 Apr 21;9:45. doi: 10.1186/s41927-025-00487-x (PMC12010607; doi:10.1186/s41927-025-00487-x)
Supplement: Supplementary file 2 — Supplementary Material 2 Supplemental Table 2: Cohort demographics and baseline clinical observations subdivided by 24-week follow-up remission status and treatment arm [file 41927_2025_487_MOESM2_ESM.docx]

**Supplemental Table 2. Cohort demographics and baseline clinical observations subdivided by 24-week follow-up remission status and treatment arm**

|  | **Rem**  MTX +  prednisolone  (n=2) | **No Rem**  MTX +  prednisolone  (n=10) | **Rem**  MTX +  anti-TNF  (n=8) | **No Rem**  MTX +  anti-TNF  (n=14) | **Rem**  MTX +  CTLA4-Ig  (n=10) | **No Rem**  MTX +  CTLA4-Ig  (n=7) | **Rem**  MTX +  Anti-IL-6R  (n=8) | **No Rem**  MTX +  Anti-IL-6R  (n=11) |
| --- | --- | --- | --- | --- | --- | --- | --- | --- |
| Age, yr^a^ | 54 (46-62) | 63 (24-80) | 59 (29-71) | 60.5 (21-71) | 56 (33-72) | 62 (21-77) | 52 (25-72) | 51 (28-64) |
| Female, n (%) | 2 (100) | 8 (80) | 5 (63) | 8 (57) | 6 (60) | 6 (86) | 6 (75) | 8 (73) |
| Smoker, n (%) ^e^ | 1 (50) | 0 (0) | 1 (13) | 3 (21) | 1 (10) | 1 (14) | 1 (13) | 3 (27) |
| Symptom duration, months^a, b^ | 4 (3-4) | 5 (1-11) | 6 (3-9) | 3 (1-18) | 4 (2-7) | 11 (3-23) | 4 (1.5-19) | 6 (1.5-21) |
| CRP, mg/L^a^ | 9.5 (4-15) | 15 (2-152) | 26 (2-68) | 16.5 (2-180) | 17 (3-92) | 5 (2-50) | 5.7 (1-22) | 5 (0.3-16) |
| ESR, mm/hr^a^ | 17 (11-23) | 30 (5-108) | 36 (11-85) | 28.5 (7-98) | 29 (10-101) | 23 (8-80) | 18.5 (7-37) | 23 (5-32) |
| SJC66^a^ | 9 (8-10) | 20 (4-30) | 18 (6-23) | 10 (3-28) | 10 (7-19) | 6 (3-18) | 12 (4-15) | 10 (3-17) |
| TJC68^a^ | 13 (12-14) | 20 (6-34) | 14 (9-25) | 16 (2-35) | 13 (8-21) | 13 (3-25) | 8.5 (3-16) | 14 (3-47) |
| SJC28^a^ | 7 (4-9) | 13 (4-22) | 13 (5-21) | 7 (2-24) | 7 (4-14) | 5 (3-10) | 9.5 (4-13) | 8 (2-12) |
| TJC28^a^ | 6.5 (4-9) | 13 (3-21) | 9 (3-14) | 7 (1-27) | 9 (4-13) | 8 (0-13) | 5.5 (2-11) | 9 (0-24) |
| DAS28-CRP^a^ | 4.3 (3.6-4.9) | 5.1 (4.7-7.7) | 5.4 (4.3-6.5) | 5.3 (3.2-8.3) | 5.4 (3.9-6.5) | 4.2 (3.8-5.2) | 4.5 (3.5-5.3) | 4.7 (2.7-6.9) |
| DAS28-ESR^a^ | 4.5 (3.7-5.2) | 5.8 (4.8-8.2) | 6.0 (4.5-6.8) | 5.4 (3.6-8.7) | 5.6 (4.2-7.2) | 5 (4.2-6.1) | 5.1 (4.1-5.6) | 5.3 (2.6-7.1) |
| CDAI^a^ | 20.6 (13.3-27.8) | 35.5 (21.6-56.9) | 34.4 (21.9-41.7) | 27.5 (10.1-68.7) | 28 (17.9-41.7) | 22.1 (14.3-33.5) | 26.8 (20.6-32.8) | 29 (10.5-52.5) |
| ACPA^+^, n (%) ^c^ | 2 (100) | 8 (80) | 7 (88) | 10 (71) | 9 (90) | 6 (86) | 5 (62.5) | 11 (100) |
| RF^+^, n (%) ^d^ | 2 (100) | 8 (80) | 5 (63) | 8 (57) | 6 (60) | 6 (86) | 6 (75) | 11 (100) |
| ACPA^+^ RF^+^, n (%) ^c, d^ | 2 (100) | 7 (70) | 5 (63) | 8 (57.1) | 6 (60) | 5 (71) | 3 (38) | 11 (100) |
| ACPA^–^ RF^–^, n (%) ^c, d^ | 0 (0) | 1 (10) | 1 (13) | 4 (28.6) | 1 (10) | 0 (0) | 0 | 0 |

a Median and range

b Retrospective patient-reported pain in joints before RA diagnosis

c Patients with ACPA levels ≥ 20IU/ml are considered ACPA+

d Patients with RF levels ≥ 20 IU/ml are considered RF+

e Current daily smoker

Rem=Remission; CRP: C Reactive Protein; ESR: Erythrocyte Sedimentation Rate; SJC: Swollen Joint Count; TJC: Tender Joint Count; DAS28: Disease Activity Score for 28 joints; CDAI: Clinical Disease Activity Index; ACPA: Anti-Citrullinated Protein antibodies; RF: Rheumatoid Factor
