# Supplementary material for: Transitional and CD21− PD-1+ B cells are associated with remission in early rheumatoid arthritis
Source: BMC Rheumatol. 2025 Apr 21;9:45. doi: 10.1186/s41927-025-00487-x (PMC12010607; doi:10.1186/s41927-025-00487-x)
Supplement: Supplementary file 3 — Supplementary Material 3 Supplemental Table 3: Demographic and clinical confounding variables with B cell populations of interest at diagnosis [file 41927_2025_487_MOESM3_ESM.docx]

**Supplemental Table 3. Demographic and clinical confounding variables with B cell populations of interest at diagnosis**

|  | B cells | Spearman’s  correlation coefficient | P-value |
| --- | --- | --- | --- |
| Age | Transitional  CD21^–^ PD-1^+^  CD21^–^ DN  CD21^–^ Pb | -0.234  -0.089  0.374  -0.082 | 0.053^e^  0.47^e^  **0.004**** ^e^  0.50^e^ |
|  |  |  |  |
| Gender | Transitional  CD21^–^ PD-1^+^  CD21^–^ DN  CD21^–^ Pb |  | 0.77^f^  0.46^f^  0.53^f^  0.34^f^ |
|  |  |  |  |
| Smoking^a^ | Transitional  CD21^–^ PD-1^+^  CD21^–^ DN  CD21^–^ Pb |  | 0.79^f^  0.34^f^  0.15^f^  0.42^f^ |
|  |  |  |  |
| Symptom duration^b^ | Transitional  CD21^–^ PD-1^+^  CD21^–^ DN  CD21^–^ Pb | -0.278  -0.036  -0.099  -0.103 | **0.021*** ^e^  0.77^e^  0.46^e^  0.40^e^ |
|  |  |  |  |
| CRP, mg/L, at diagnosis | Transitional  CD21^–^ PD-1^+^  CD21^–^ DN  CD21^–^ Pb | 0.041  -0.083  0.028  0.077 | 0.74^e^  0.50^e^  0,84^e^  0.53^e^ |
|  |  |  |  |
| 24-week CRP, mg/L, all treatment arms | Transitional  CD21^–^ PD-1^+^  CD21^–^ DN  CD21^–^ Pb | -0.006  -0.203  -0.02  -0.1 | 0.96^e^  0.09^e^  0.88^e^  0.41^e^ |
|  |  |  |  |
| 24-week CRP, mg/L, anti-IL-6R excluded | Transitional  CD21^–^ PD-1^+^  CD21^–^ DN  CD21^–^ Pb | -0.136  -0.148  -0.032  -0.17 | 0.35^e^  0.30^e^  0,84^e^  0.24^e^ |
|  |  |  |  |
| ESR mm/hr, at diagnosis | Transitional  CD21^–^ PD-1^+^  CD21^–^ DN  CD21^–^ Pb | 0.067  -0.164  0.078  0.145 | 0.58^e^  0.17^e^  0.56^e^  0.24^e^ |
|  |  |  |  |
| 24-week ESR mm/hr | Transitional  CD21^–^ PD-1^+^  CD21^–^ DN  CD21^–^ Pb | 0.009  -0.106  0.063  0.004 | 0.94^e^  0.38^e^  0.64^e^  0.98^e^ |
|  |  |  |  |
| ACPA^+ c^ | Transitional  CD21^–^ PD-1^+^  CD21^–^ DN  CD21^–^ Pb |  | 0.64^f^  0.28^f^  0.18^f^  0.60^f^ |
|  |  |  |  |
| RF^+ d^ | Transitional  CD21^–^ PD-1^+^  CD21^–^ DN  CD21^–^ Pb |  | 0.47^f^  0.18^f^  **0.03*** ^f^  0.18^f^ |
|  |  |  |  |
| ACPA^+^ RF^+^ | Transitional  CD21^–^ PD-1^+^  CD21^–^ DN  CD21^–^ Pb |  | 0.46^f^  0.32^f^  **0.02***^f^  0.77^f^ |
|  |  |  |  |
| ACPA^–^ RF^–^ | Transitional  CD21^–^ PD-1^+^  CD21^–^ DN  CD21^–^ Pb |  | 0.48^f^  0.34^f^  0.18^f^  0.43^f^ |

a Current daily smoker

b Retrospective patient-reported pain in joints before diagnosis of rheumatoid arthritis

c Patients with ACPA levels ≥ 20IU/ml are considered ACPA^+^

d Patients with RF levels ≥ 20 IU/ml are considered RF^+^

e Spearman’s Rank test, *p< 0.05, ** P<0.01

f Mann-Whitney U-test, *p< 0.05

CRP: C Reactive Protein; ESR: Erythrocyte Sedimentation Rate; ACPA: Anti-Citrullinated Protein antibodies; RF: Rheumatoid Factor; Pb: Plasmablast; DN: Double Negative
