# Supplementary material for: Transitional and CD21− PD-1+ B cells are associated with remission in early rheumatoid arthritis
Source: BMC Rheumatol. 2025 Apr 21;9:45. doi: 10.1186/s41927-025-00487-x (PMC12010607; doi:10.1186/s41927-025-00487-x)
Supplement: Supplementary file 4 — Supplementary Material 4 Supplemental Table 4: Demographic and clinical confounding variables association with treatment response [file 41927_2025_487_MOESM4_ESM.docx]

**Supplemental Table 4. Demographic and clinical confounding variables association with treatment response**

|  | Outcome  Week 24 | Spearman’s  correlation coefficient | P-value |
| --- | --- | --- | --- |
| Age | Remission  CDAI | 0.152 | 0.73^d^  0.21^e^ |
|  |  |  |  |
| Symptom duration^a^ | Remission  CDAI | 0.154 | 0.44^d^  0.20^e^ |
|  |  |  |  |
| RF^+ b^ | Remission  CDAI |  | 0.31^f^  0.4^d^ |
|  |  |  |  |
| ACPA^+^RF^+ c^ | Remission  CDAI |  | 0.15 ^f^  0.23^d^ |
|  |  |  |  |
| CRP, at diagnosis, all patients | Remission  CDAI | -0.60 | 0.67^d^  0.62^e^ |
| CRP, at diagnosis, anti-IL-6R excluded | Remission  CDAI | -0.037 | 0.61^d^  0.80^e^ |

a Retrospective patient-reported pain in joints before diagnosis of rheumatoid arthritis

b Patients with RF levels ≥ 20 IU/ml are considered RF^+^

c Patients with ACPA levels ≥ 20IU/ml are considered ACPA^+^

d Mann-Whitney U-test

e Spearman’s Rank test

f Chi-Square test

CDAI: Clinical Disease Activity Index; ACPA: Anti-Citrullinated Protein Antibodies; RF: Rheumatoid Factor; CRP: C Reactive Protein
