# Supplementary material for: Transitional and CD21− PD-1+ B cells are associated with remission in early rheumatoid arthritis
Source: BMC Rheumatol. 2025 Apr 21;9:45. doi: 10.1186/s41927-025-00487-x (PMC12010607; doi:10.1186/s41927-025-00487-x)
Supplement: Supplementary file 5 — Supplementary Material 5 Supplemental Table 5: Statistical analysis of transitional B Cells in eRA patients at diagnosis across treatments [file 41927_2025_487_MOESM5_ESM.docx]

| Kruskal-Wallis test, P value = 0,7566 |  |  |
| --- | --- | --- |
| Dunn's multiple comparisons test | Mean rank diff, | Adjusted P Value |
| MTX and Prednisolone vs. MTX and anti-TNF | -5,242 | >0,9999 |
| MTX and Prednisolone vs. MTX and CTLA4-Ig | -3,833 | >0,9999 |
| MTX and Prednisolone vs. MTX and anti-IL-6R | 0,8246 | >0,9999 |
| MTX and anti-TNF vs. MTX and CTLA4-Ig | 1,409 | >0,9999 |
| MTX and anti-TNF vs. MTX and anti-IL-6R | 6,067 | >0,9999 |
| MTX and CTLA4-Ig vs. MTX and anti-IL-6R | 4,658 | >0,9999 |

**Supplemental Table 5. Statistical analysis of transitional B Cells in eRA patients at diagnosis across treatments**

MTX: Methotrexate; anti-TNF: (certolizumab-pegol); CTLA4-Ig (abatacept); anti-IL-6R: (tocilizumab).
