# Supplementary material for: Transitional and CD21− PD-1+ B cells are associated with remission in early rheumatoid arthritis
Source: BMC Rheumatol. 2025 Apr 21;9:45. doi: 10.1186/s41927-025-00487-x (PMC12010607; doi:10.1186/s41927-025-00487-x)
Supplement: Supplementary file 6 — Supplementary Material 6. Supplementary Figures [file 41927_2025_487_MOESM6_ESM.docx]

***
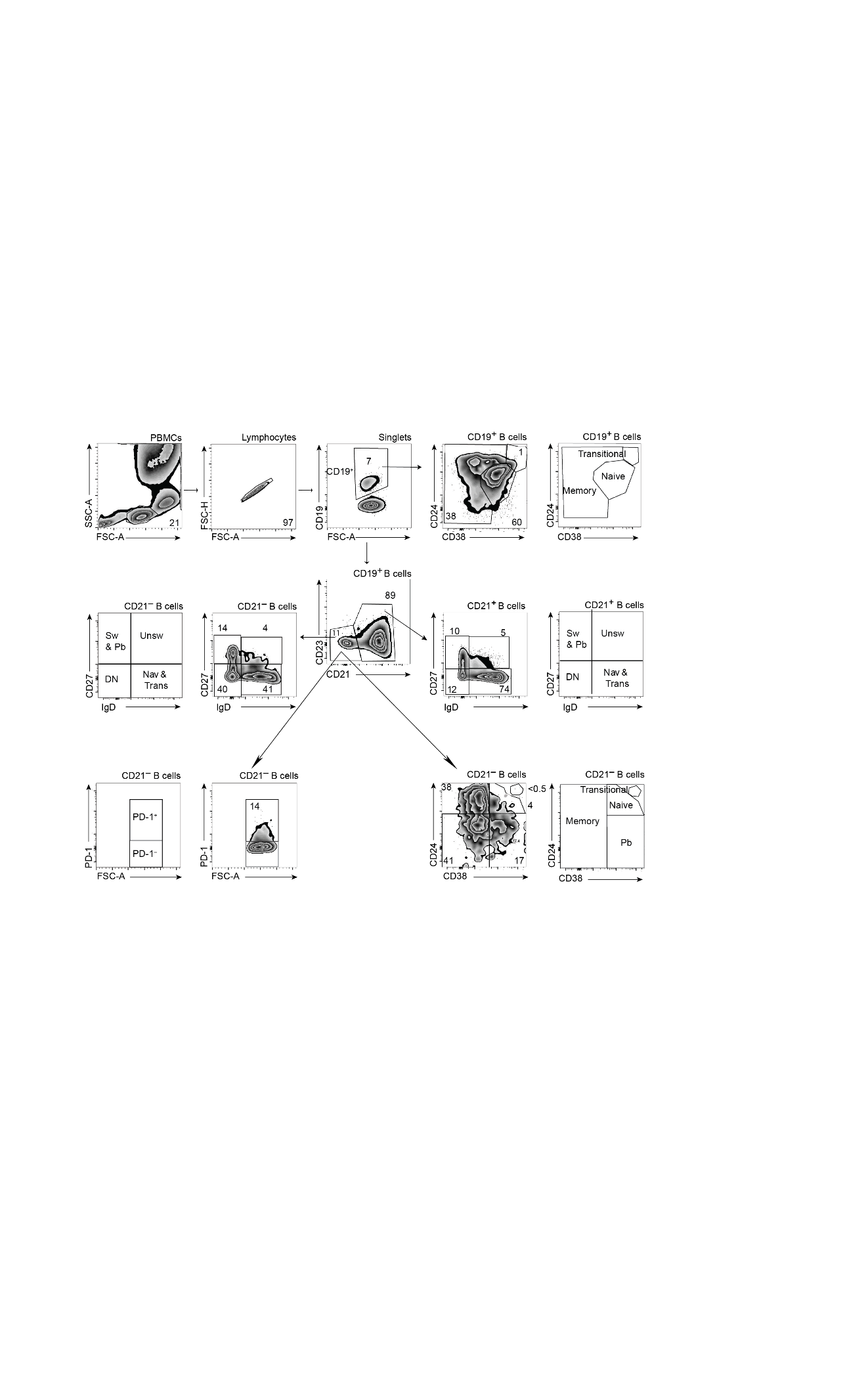
***

***Supplementary figure 1: Gating strategy to define circulating B-cell subsets.***

*Circulating B cells were identified in PBMCs as single lymphocytes expressing CD19. Co-expression of CD24 and CD38 within CD19^+^ B cells was used to determine transitional B cells (CD24^++^ CD38^++^), naive B cells (CD24^+^ CD38^+^), and memory B cells (CD24^+/lo^ CD38^lo^). CD19^+^ B cells were also divided according to their expression of the CD21 coreceptor into CD21^+^ and CD21^–^ populations. Gating on CD27 vs IgD, we defined a further four B cell subsets: naive and transitional cells (Nav, Trans, CD27^–^ IgD^+^), switched MBCs and plasmablast (Sw, Pb, CD27^+^ IgD^–^), unswitched MBCs (UnSw, CD27^+^ IgD^+^), and double negative MBCs (DN, CD27^–^ IgD^–^); these populations were identifiable in both CD21^+^ and CD21^–^ populations. Within the CD21^–^ population plasmablasts were identified as CD38^++^, CD24^–/lo^ and the CD21^–^ B cells were also divided according to their expression of PD-1.*

***
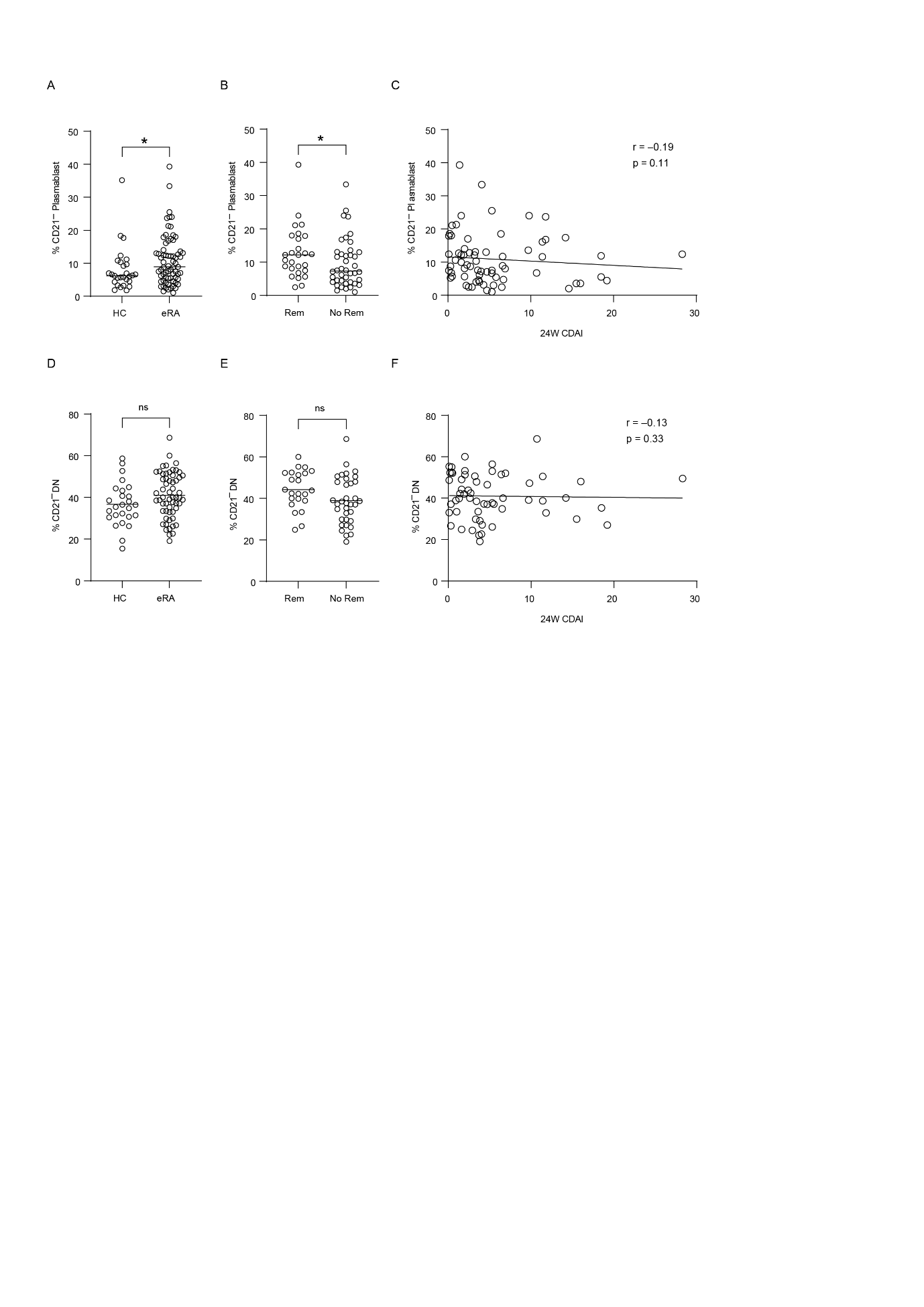
***

***Supplementary figure 2: CD21^–^ Pb and CD21^–^ DN B cells in eRA at diagnosis and association with 24-week remission.***

*(A) Frequency of Pb (of parent population CD21^–^ B cells) in HC (N=28) and in eRA patients (N=69) at diagnosis, Mann-Whitney U-test. (B) Frequency of Pb, in eRA patients at diagnosis, subdivided by 24-week follow-up remission status, Mann-Whitney U-test. (C) Scatter plot and Spearman’s Rank correlation analyses of CD21^–^ Pb frequency at diagnosis against 24-week follow-up CDAI. (D) Frequency of DN (of parent population CD21^–^ B cells) in HC (N=26) and in eRA patients (N=58) at diagnosis, unpaired T-test. (E) Frequency of CD21^–^ DN, in eRA patients at diagnosis, subdivided by 24-week follow-up remission status, unpaired T-test. (F) Scatter plot and Spearman’s Rank correlation analyses of CD21^–^ DN frequency at diagnosis against 24-week follow-up CDAI. In panels A and B, horizontal lines indicate the median values, while in panels D and E, horizontal lines indicate the mean values. *p< 0.05; ns: non-significant; Pb: Plasmablast; DN: Double Negative. HC: Healthy Control; eRA: early Rheumatoid Arthritis; Rem: Remission; CDAI: Clinical Disease Activity Index*


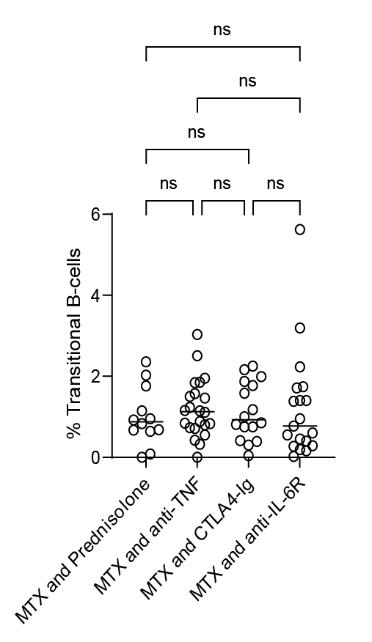


***Supplementary figure 3: Frequencies of transitional B cells in eRA patients at diagnosis across treatments.***

*Frequency of transitional B cells (CD24^++^CD38^++^; in total B cells) in eRA patients (N=69) at diagnosis, subdivided by treatment arm; MTX and Prednisolone (N=12), MTX and anti-TNF (N=22), MTX and CTLA4-Ig (N=16), MTX and anti-IL-6R (N=19), Kruskal-Wallis test followed by Dunn's multiple comparisons test. Horizontal lines indicate the median value. ns: non-significant; MTX: Methotrexate; anti-TNF: (certolizumab-pegol); CTLA4-Ig (abatacept); anti-IL-6R: (tocilizumab).*

***
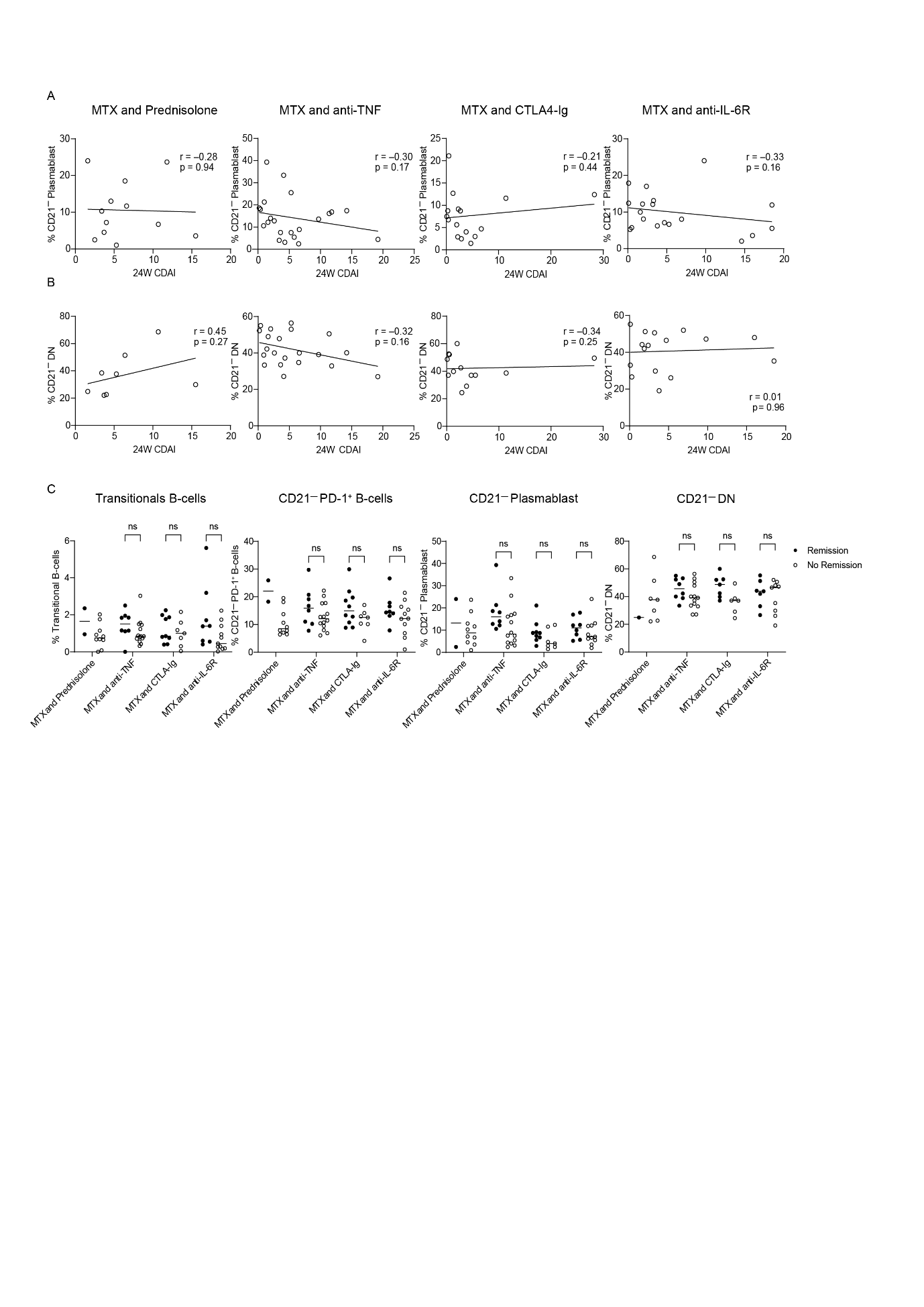
***

***Supplementary figure 4: B-cell subpopulations in eRA at diagnosis and association with 24-week CDAI and remission divided by treatment arm***

*(A) Scatter plot and Spearman’s Rank correlation analyses of Pb (of parent population CD21^–^ B cells) frequency at diagnosis against 24-week follow-up CDAI in the following treatment arm; MTX and Prednisolone (N=12), MTX and anti-TNF (N=22), MTX and CTLA4-Ig (N=16), MTX and anti-IL-6R (N=19). (B) Scatter plot and Spearman’s Rank correlation analyses of DN B-cells (of parent population CD21^–^ B cells) frequency at diagnosis against 24-week follow-up CDAI in the following treatment arms; MTX and Prednisolone (N=8), MTX and anti-TNF (N=21), MTX and CTLA4-Ig (N=13), MTX and anti-IL-6R (N=16). (C) Frequency of transitional B cells (CD24^++^CD38^++^; in total B cells), CD21^–^ PD-1^+^ B-cell (of parent population CD21^–^ B cells), Pb and CD21^–^ DN at diagnosis, subdivided by 24-week remission status and treatment arm, Mann-Whitney U-test followed by Bonferroni-Dunn correction for multiple tests. Horizontal lines indicate the median value; ns: non-significant. DN: Double Negative; Pb: Plasmablast; eRA: early rheumatoid arthritis; MTX: Methotrexate; anti-TNF: (certolizumab-pegol); CTLA4-Ig (abatacept); anti-IL-6R: (tocilizumab); CDAI: Clinical Disease Activity Index.*
